# Health knowledge and livelihood experiences with COVID-19 amongst Arizona residents

**DOI:** 10.3389/fpubh.2022.939154

**Published:** 2022-10-17

**Authors:** Tina Fingesi, Lin Chung Yon, Sheila Soto, Cecilia Rosales

**Affiliations:** ^1^Community, Environment and Policy, Mel and Enid Zuckerman College of Public Health, University of Arizona, Tucson, AZ, United States; ^2^College of Medicine, University of Arizona, Phoenix, AZ, United States; ^3^Public Health Practice and Translational Research, Mel and Enid Zuckerman College of Public Health, University of Arizona, Phoenix, AZ, United States

**Keywords:** COVID-19, health disparities, Hispanic, marginalized communities, Arizona

## Abstract

The Coronavirus disease 2019 (COVID-19) pandemic is an ongoing public health concern that is rapidly evolving and has impacted individuals and communities differently. We analyzed deidentified survey datasets to evaluate the perceptions, experiences, and impacts of COVID-19 among Arizona residents. The survey included 1,472 eligible Spanish-speaking participants in Southern (Pima, Santa Cruz, Cochise, Yuma County) and Central Arizona (Maricopa County). Eighteen questions which included participants' health and socio-economic status, source of information on COVID-19, preventive measures, the impact of COVID-19 on household income, and vaccination status were administered to the survey respondents. The analyzed data showed an unequal proportion of the reported source of COVID-19 information between Southern and Central Arizona participants. More male respondents (*n* = 833, 57%) participated in the study than did the female respondents (*n* = 638, 43%). Of the 1,472 total participants in both regions, 1,011 (68.7%) participants represented Southern Arizona while 461 (31.3%) participants represented Central Arizona. Of the 461 participants in Central Arizona, the majority reported television (56%) and social media (20%) as their primary source of information. Whereas, of the 1,011 participants in Southern Arizona, the majority reported social media (37%) and television (32%) as their major source of information on COVID-19. Overall, 82% of the participants were vaccinated, with a statistically significant difference between the proportion of vaccinated individuals in the Southern and Central Arizona (chi-square *p*-value of 0.00139). More individuals in Southern Arizona participated in the survey than in Central Arizona across both genders, with 58% of women reporting loss of jobs due to COVID-19. This study demonstrated that the COVID-19 pandemic profoundly had a more socio-economic impact on women than men, particularly Hispanic women in this subset.

## Introduction

The impact of Coronavirus disease 2019 (COVID-19) on health and well-being varies across communities. COVID-19 has exacerbated existing structural and social inequalities, with particularly undesirable health outcomes for those already disadvantaged in the society (1). After nearly 3 years, many individuals and families still face persisting limitations to secure means of livelihood, as a result of the pandemic. Specific groups are striving for basic amenities and medical treatment (2), this contributes to the feelings of inequality, discrimination, and isolation among marginalized communities (3). The knowledge of COVID-19 is evolving daily, consequently, society encounters diverse forms of contradictory information, incomplete information, and sometimes outright misinformation (4). People still tend to prefer informal source like social media or family and friends as their primary source of information, therefore, it is now more imperative than ever to evaluate the basic health knowledge of the public on COVID-19 (5).

Health literacy improves health and well-being, addresses health inequalities, and builds individual and community resilience, allowing individuals to make better health decisions and have a stronger commitment and higher levels of efficiency (6). Since the onset of the COVID-19 pandemic, there have been numerous publicly available data from legitimate and illegitimate source (7), this overflow of information gathering makes it difficult for the public to decipher the accurate information from misleading information, resulting in misconceptions and wrong beliefs. The internet has been associated with lower literacy levels (8) due to the amount of fake information disseminated without technical review and appraisal (9). Although young adults may have high digital health literacy about COVID-19, discerning the reliability of this online health information may be challenging. Online communication channels are especially vulnerable to the spread of incorrect information making people adopt wrong behaviors against COVID-19 (5). However, online communication channels have also been a central resource for reliable health information throughout the pandemic.

Numerous pre-pandemic disparities unfolded during COVID-19 with communities of color suffering disproportionately (10). Before the pandemic, many families, especially those with lower incomes, faced significant difficulty in their economic, physical, and mental well-being. In addition, the COVID-19 pandemic has led to an immense economic and public health disruption, amplifying previously existing economic inequalities; and disproportionately affecting Black and Hispanic workers, women, young adults, and people with low incomes. Some communities can withstand the impact of economic downturns due to more favorable economic and social factors protecting residents from adversity. However, other communities are experiencing the effect of rising unemployment and financial troubles during the time of COVID-19. The loss of income and livelihood has further led to drops in wages, pressuring more people into poverty, which simultaneously impacts community health (11).

Obtaining a health coverage plays a major role in determining access to health care amongst People of Color (12). There were early reports of racial disparities when the COVID-19 vaccines were in short supply relative to its demand (13). Despite the COVID-19 vaccines supply exceeded demand at the time of this study, racial disparities in vaccination were still apparent. Upstream social determinants of health are accounting for the vaccine disparity, including disproportionate access to vaccines in low-income neighborhoods, inability to skip work to receive a vaccine, lack of access to culturally and linguistically tailored information, and fear of deportation among some immigrant groups (14–17). These upstream social determinants are evident in previous vaccination programs, where racial and ethnic minority groups experienced persistent low annual influenza vaccination rates compared to White persons (18).

The ease of restrictions and returns to normalcy can be attributed to the increase in the number of vaccinated populations. While innovative and useful research continues to emerge, additional information and analysis of robust data source will help policymakers better understand how the pandemic has disproportionately affected populations that have historically faced barriers to accessing health services (19). In Arizona, persons living in poverty are disproportionately American Indians, Hispanic, and Black, with Hispanic persons more often reporting lack of healthcare coverage or not visiting a doctor because of costs (20). We attempt to fill this gap by focusing the Mobile Health Unit (MHU) vaccination and survey questions to the hardly reached populations in Arizona, represented by ethnic minorities, migrant farmworkers, the elderly, people without homes, undocumented immigrants amongst others. To evaluate the perception, experiences, and impacts of COVID-19, we analyzed deidentified survey datasets with inquiries about participants' primary source of information, change in employment status, and access to COVID-19 vaccination among Arizona residents.

## Methodology

The Mobile Outreach Vaccination & Education for Underserved Populations-Mobile Health Units (MOVE-UP MHUs) provide vaccination, free preventative health screenings, health education, and other health-related services to vulnerable, hard-to-reach populations across Southern and Central Arizona. However, from March-April 2020 during the initial lockdown and business closure due to the COVID-19 pandemic, the MHU did not provide on-site services and instead initiated a call-back campaign to previous users. In the late fall of 2020, the MHUs carefully reinitiated on-site visits and shortly after, the MOVE-UP project commenced. The MHU staff provided credible health information regarding COVID-19, emotional support, and serve as a link to community resources during a difficult time. The method was later synchronized and used by the Mexican Section of the U.S.-Mexico Border Health Commission across all 11 mobile health units (21) and 50 Ventanillas de Salud (VDS) and (Health Windows (HW) inside Mexico's Consular Network across the United States (22). Due to the high-need responses during the call-back campaign, the MHU staff provided mobile unit users with a paper version of the “COVID-19 call-back survey.” The MOVE-UP project was later created to help increase COVID-19 vaccination rates in Arizona with an emphasis among vulnerable populations. The project allowed for the MHU to expand its reach and go directly to areas with low vaccination or low access to points of COVID-19 vaccine distribution.

The survey included 1,472 eligible Spanish-speaking participants in Southern (Pima, Santa Cruz, Cochise, Yuma County) and Central Arizona (Maricopa County). In the southern area of study, Pima is a large county classified as a medium metro area, Santa Cruz is a small county classified as a micropolitan or large rural area, Cochise and Yuma are both medium counties classified as small metro area. While Maricopa is the largest county classified as a large central metro area; according to the National Center for Health Statistics Urban-Rural Classification (“Arizona Health Workforce Profile”)^1^. The Central (urban) and Southern (rural) Arizona regions are of significance to this study due to the significant gap between numbers of health professionals in the urban and the rural areas of Arizona (“Arizona Health Workforce Profile”)^1^. In addition to the southern region having more rural areas, it is also closer to the border than the central region. Moreso, the “mobile health units” are primarily housed in these two regions. The mobile health units were strategically placed in target cities based on the concentration of Hispanic population determined by the network of consular offices in the U.S. and the Mexico Section of the U.S. Mexico Border Health Commission (USMBHC). The Arizona health workforce profile shows that health care infrastructure and workforce are more concentrated in metro areas of Arizona than the rural areas (23).

The administered survey focused on questions pertaining to participants' socio-economic and health status, primary source of information on COVID-19, implemented preventive measures, the impact of COVID-19 on household income, and current vaccination status (Appendix 1). Gender in this study is an important variable that was measured from the respondents' responses, each participant self-identified to one of the three gender options: male, female, or transgender. The authors used de-identified data from a database system called the Continuous Information System and Health Reports of Mexicans in the United States (SICRESAL-MX [acronym in Spanish]) to perform this secondary analysis. SICRESAL-MX is a computer-based system developed by the Mexican Section of the USMBHC, specifically to confidentially maintain information provided by users in the HW and MHU's. The use of secondary data for this analysis was not deemed human subjects research, therefore, did not require IRB approval. This study excluded incomplete data from phone or in-person survey responses.

### Statistical analysis

The results collected from the questionnaires were analyzed using RStudio “Prairie Trillium” Release with R version 4.1.3, and the graphs were generated with the ggplot2, ggpubr, and ggsci packages. The chi-square test was conducted with the chisq.test function within R. We removed questionnaires with blank entries pertinent to the test before generating the summary statistics and chi-square tests. Overall, the observed numbers of missing data can be attributed to the design of the study being a voluntary survey, where participants can consent to partake in the study with the will to answer or omit any question, without affecting their participation in the survey. For the bivariate analyses, we investigated how female and male respondents in the Central and Southern Arizona responded to questions such as “Have you received the first dose of COVID-19 vaccine?,” “Did you lose your job during the pandemic?,” and “Do you know any family member who had experienced job loss during the pandemic?.” The R script and data used to generate the results to this publication are available upon request.

## Result

A total of 1,472 eligible respondents participated in the survey, male respondents represented the higher proportion (*n* = 833, 57%) with an average age of 39 (±18) years old across both genders, while female respondents represented 43% (*n* = 638) of the participants. A larger sum of the participants (59%) identified as Mexicans, as opposed to 10% identifying as United States nationalities. This can be attributed to why more participants (805) omitted the responses to the English fluency question compared to the Spanish fluency question (302). The distribution of the sample analysis is shown in Table 1.

**Table 1:** Social and demographic characteristics of respondents.

**Variable**	**Subgroups**	**Frequency (%)**
Age (years)*	< 18	61 (4.1)
Mean ± SD: 39 ± 18 Median: 39 IQR: 17	18–24	146 (9.9)
	25–44	795 (54.0)
	45–64	408 (27.7)
	> 65	62 (4.2)
Gender	Female	638 (43.0)
	Male	833 (57.0)
	Transgender	1 (0.0)
English fluency	Not at all	117 (6.9)
	Not well	82 (4.8)
	Well	60 (3.5)
	Very well	629 (37.2)
	Missing data	805 (47.6)
Spanish fluency	Not at all	7 (1.5)
	Well	17 (3.7)
	Very well	135 (29.2)
	Missing data	302 (65.5)
Birth country	United States	46 (9.9)
	Mexico	273 (59.2)
	Guatemala	27 (5.9)
	Nicaragua	1 (0.2)
	El Salvador	3 (0.6)
	Venezuela	1 (0.2)
	Chile	1 (0.2)
	Peru	1 (0.2)
	Cuba	2 (0.4)
	Honduras	2 (0.4)
	Missing data	104 (22.6)
State region of participants	Southern Arizona	1,011 (68.7)
	Central Arizona	461 (31.3)
Lives with family	Yes	251 (54.4)
	No	210 (45.6)
Medical insurance	Yes	95 (20.6)
	No	366 (79.3)
Educational status	None	11 (2.4)
	Elementary school (5th grade)	10 (2.2)
	Middle school (6th−8th grade)	31 (6.7)
	High school (9th−12th grade)	80 (17.4)
	Some years of university	13 (2.8)
	Finished University	11 (2.4)
	Postgraduate	1 (0.2)
	Missing data	304 (65.9)
Total household income	Does not know	14 (3.0)
	Less than 1,000 USD	60 (13.0)
	Between 1,000 USD and $3,000 USD	78 (16.9)
	Between 3,001 USD and $5,000 USD	1 (0.2)
	Refused	4 (0.8)
	Missing data	304 (65.9)

^*^MEPS age grouping.

**Figure 1:**
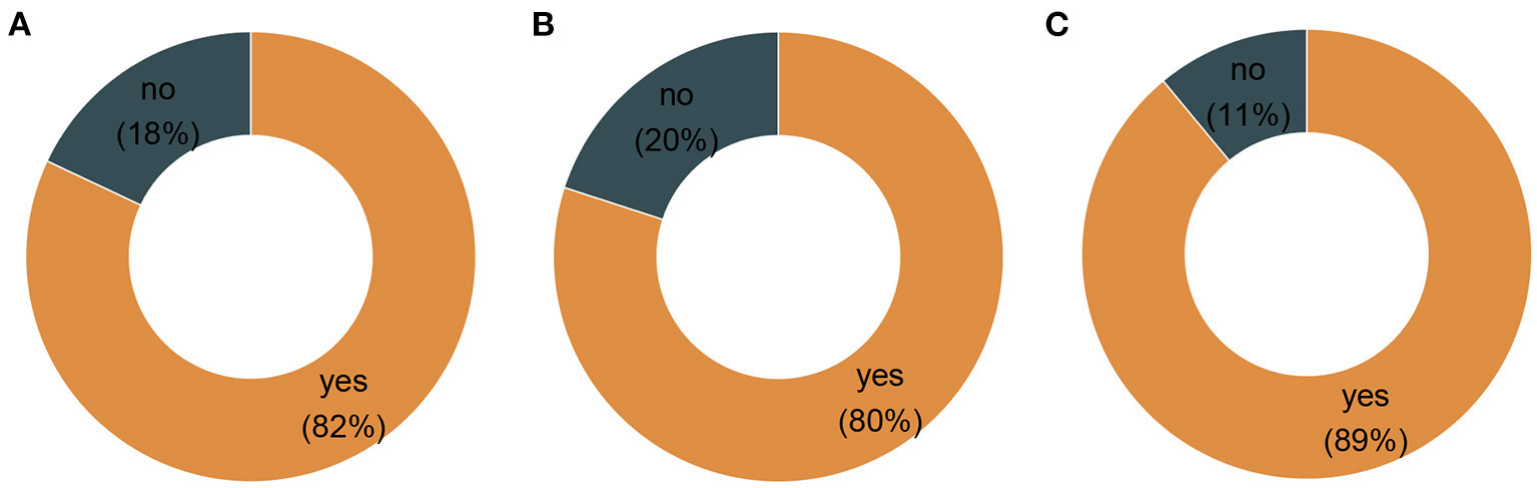
Proportions of vaccination per region of the study area. **(A)** Combined region vaccination rate. **(B)** Southern Arizona vaccination rate. **(C)** Central Arizona vaccination rate.

There were more participants across both genders in Southern Arizona (69%) (Pima, Santa Cruz, Cochise, Yuma) than in Central Arizona (31%) (Maricopa). Since the survey administration, 82% of the participants in the study had received a COVID-19 vaccine (Figure 1A), with a statistically significant difference between the proportion of vaccinated individuals in the Southern Arizona (Figure 1B) and Central Arizona (Figure 1C) (chi-square *p*-value of 0.00139). We investigated the rate of vaccination and found that residing in Central Arizona (Maricopa County) was associated with a significantly higher proportion of vaccination among MHU users (chi-sq =10.8, *p* < 0.05), when compared to the Southern Arizona cohort.

**Figure 2:**
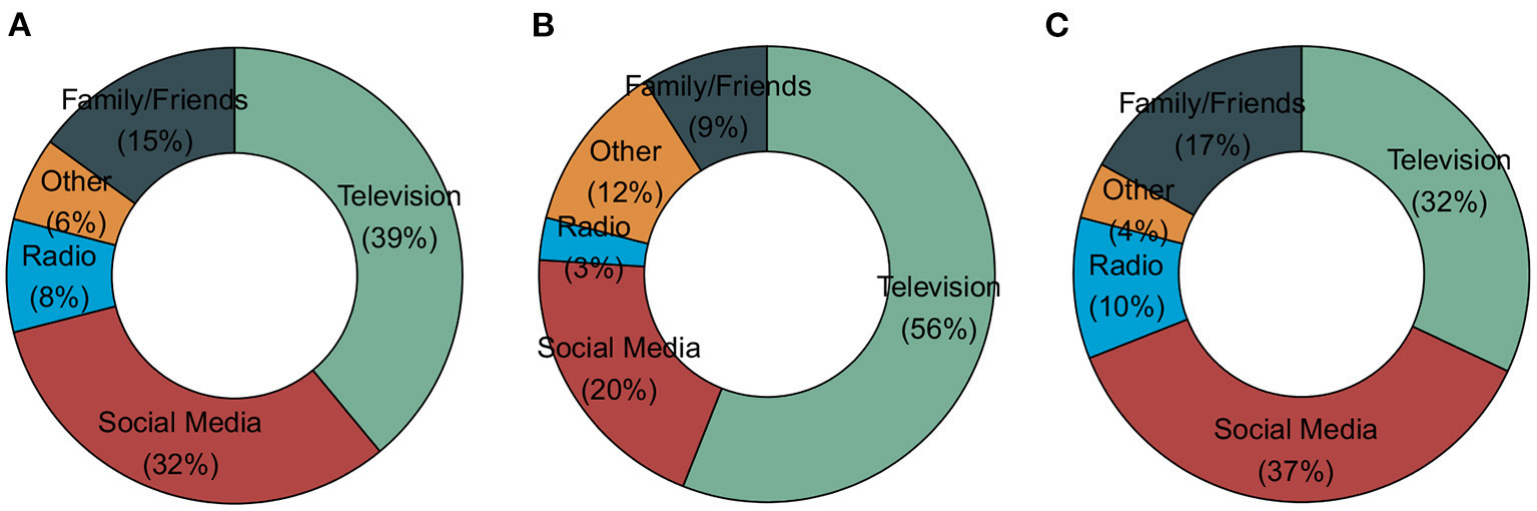
COVID-19 information source. **(A)** Combined region information source. **(B)** Central Arizona information source. **(C)** Southern Arizona information source.

There was an unequal proportion of the reported source of COVID-19 information between Southern Arizona and Central Arizona (Figure 2). Upon further investigation, we found that of the 461 (31%) participants in Maricopa County, 56% of the respondents reported a higher proportion of television as their primary source of information when compared to the Southern Arizona cohort with a higher proportion reporting social media (37%) as the primary source of information.

A significantly higher proportion of female respondents (86%) received at least one dose of the COVID-19 vaccine compared to males (80%, *x*^2^ = 7.66, *p* < 0.05). Furthermore, a significantly higher proportion of female respondents (58%) reported job loss during the pandemic than male respondents (42%, *x*^2^ = 6.54, *p* < 0.05). More female respondents (17%) also recorded having a family member who had experienced job loss during the pandemic than male respondents (11%, *x*^2^ = 7.48, *p* < 0.05). This study demonstrated that the COVID-19 pandemic had a more profound socio-economic impact on women than men, particularly Hispanic women.

## Discussion

During the COVID-19 pandemic, a vast amount of information has been readily available through the media, internet, social networks, and many other sources (5). The way people access information has changed over the last decade, with younger generations tending to rely on the internet as their preferred source of information. Overall, the majority of the participants in this survey were in the age group of 19–40 years and most specified television and social media as their primary source of information, with a preference for television (56%) over social media (20%) from the central/metro region. Health literacy and knowledge has been shown to influence preventive behavior among chronic disease patients (24), as improvements in health literacy are likely to result in improved utilization of preventive services, medical adherence, and involvement in health decision-making (25).

Although the southern region specified television and social media as well, they preferred social media (37%) over television (32%). The southern region consists more of counties classified as rural areas (Yuma, Cochise, Santa Cruz), with the exception of Pima county which also expands into areas close to the US-Mexico border. On account of income and poverty rate, these factors may be a contributing influence on why those in urban areas may be more socially advantaged to afford a television than the rural populations. Phoenix in central Arizona for instance has a lower poverty rate of 16.2%, a median household income of $60,914 and an employed population of about 822,717; whereas, Tucson in Southern Arizona has a higher poverty rate 20.8%; a lower median household income of $45,227 and a lower employed population of 249,855 relative to the metro central Arizona (SVI CDC)^2^.

In spite of the central region having some rural areas, it is predominantly an urban area. The social vulnerability index (SVI) is another factor that could account for why the central region (Maricopa SVI of 0.6354 which denotes moderate vulnerability) seem to be more socially advantaged than the southern region: Yuma SVI of 0.9895, Cochise SVI of 0.9064, Pima SVI of 0.8828 and Santa Cruz SVI of 0.9318, all indicating a high level of vulnerability (26). The greatest impact of COVID-19 was in rural areas where residents tend to be older (23), and workers are mostly essential workers that are unable to work from home (27). Report findings showed that there are fewer health providers per 100,000 population in rural than urban areas of Arizona with County health departments reporting difficulties providing services due to budget cuts and problems with hiring and retaining health staff primarily due to uncompetitive wages (27).

Our study revealed that a higher proportion of the respondents (82%), with the help from the MOVE-UP project had received a COVID-19 vaccination when this survey was conducted, which further reiterated the success of this vaccination program. In this study, women reported more vaccination tendencies than men, and these findings resonate with a survey that identified gender differences in health and the use of health services to be a long-standing concern for the U.S. medical system. Such differences have been documented in physician and home care use, hospital service, outpatient surgery, and preventive services (28). A study investigating COVID-19 vaccine hesitancy across geographic patterns in the United States found that men (36%) were more likely than women (22%) to cite personal reasons for not taking a vaccine (29). This coincides with another study that showed that women constitute the majority of Medicare beneficiaries (28).

Majority of the vaccinated participants in this study received their COVID-19 vaccines through the Mobile Outreach Vaccination & Education for Underserved Populations (MOVE-UP) in Arizona -an initiative developed to expedite vaccinations to vulnerable, hard-to-reach individuals within the state (30); as predicted, that access to healthcare service may be difficult for racial and ethnic minorities. Community efforts such as this led to improved results in the early days of COVID-19 vaccination. A CDC report found that Arizona was as at the time of its report one of only two states (Arizona and Montana) that had greater COVID-19 vaccination coverage in its counties with high social vulnerability than its counties with low social vulnerability across all metrics, according to the social vulnerability index (31). Current research findings show that Hispanic and Black households report disproportionate loss of income from the pandemic, with approximately 70% of Hispanic households reporting an income shock during the pandemic as of July 2020, compared to 60% of Black households and 50% of non-Hispanic White households (32). Without sufficient support, families of color stand at risk of experiencing significant percentage declines in wealth due to the COVID-19 recession, as they did as a result of the Great Recession (33).

Notably, women and people of color, specifically Black and Hispanic workers, are overrepresented in the low-wage workforce (34). In this study, female respondents reported a more socio-economic impact during the pandemic by reporting higher job loss than male respondents. Efforts to address the long-term impacts of the COVID-19 pandemic on economic well-being should prioritize an equitable recovery (19). Community engagement and targeted interventions can help identify and lessen the root causes of the disparities, such as the social determinants of health and pre-existing comorbidities (35). The immediate disproportionate impact of the COVID-19 pandemic on racial and ethnic minority groups with insufficient resources is receiving more awareness (36). However, insufficient funding, or the lack thereof, to support the research or interventions of under-represented minority groups often leads research investigators to change research interests and channel their research focus to other competitive topics, away from health disparities in minority groups. Many scientists from under-represented minority groups focus their research on projects that address the needs of their communities, which may not be well aligned with the strategic priorities of their institutions or funding agencies, urging grant reviewers to question the significance of focusing research on minority populations, if the health disparities are not glaringly obvious (37). Therefore, it is pertinent to reexamine policy interventions in order to redress the disproportionate burdens and lack of resources for ethnic minority groups.

The outcomes of this study present some of the socio-economic impacts of the COVID-19 pandemic and the persistent disparities among minority populations. A more inclusive access begins with the availability of accurate information for all. Therefore, to reduce some common barriers to effective communication in a pandemic, one of the inching steps implied by these findings; is for academia, public health experts, healthcare institutions, clinicians, and federal government agencies to re-examine current programs. Such that the dissemination of updated information with reference to the public is not limited to white papers, scientific papers published in journals and other similar scholarly articles, but also channeled through informal digital platforms such as television and social media which are more easily accessible for minority groups, as shown from participants' responses in this study.

This article has two main limitations. First is selection bias, due to the ease of data collection, geographical proximity, availability, and willingness to participate in the study during mobile unit visits, the participants were recruited through convenience sampling, however this may not be representative of the population of interest. Secondly, there was a huge amount of missing data for important variables such as education, income, language, and nationality as responses to any and all questions was to the discretion of the participant. Despite these limitations, this study yielded several useful insights about the population-specific health disparities experienced during the COVID-19 pandemic, as well as elucidated the disproportionate socio-economic impact COVID-19 had on residents across the study area in Arizona. This survey presents valuable information for policy makers such as Arizona Public Health Association, Arizona Department of Health Services, Arizona Medical Association Advocacy, and other community healthcare service providers. This result can be used as a basis to realign current programs and outreach in an effort to incorporate equity into the many health policy decisions made yearly, enhancing the health professions training pipeline to include training in rural areas, addressing scope of practice regulations to promote practice in rural areas (27). Such successful models can then be replicated and modified in other states, and among local government agencies and non-profit organizations, which will aid a gradual ease of the burden of health inequalities and disparities amongst populations of diverse groups, toward a near-equitable positioning in a future pandemic.

## Data availability statement

The raw data supporting the conclusions of this article will be made available by the authors, without undue reservation.

## Author contributions

CR contributed to the conception and design of the study. SS organized the database and proofread sections of the manuscript with corrections. LC performed the statistical analysis. TF wrote the first draft of the manuscript. All authors contributed to manuscript revision, read, and approved the submitted version.

## Conflict of interest

The authors declare that the research was conducted in the absence of any commercial or financial relationships that could be construed as a potential conflict of interest.

## Publisher's note

All claims expressed in this article are solely those of the authors and do not necessarily represent those of their affiliated organizations, or those of the publisher, the editors and the reviewers. Any product that may be evaluated in this article, or claim that may be made by its manufacturer, is not guaranteed or endorsed by the publisher.

## Footnotes

^1^ https://crh.arizona.edu/sites/default/files/2022-03/20210420_CriticalCareWorkforceBrief.pdf

^2^ https://svi.cdc.gov/map.html
