# Supplementary material for: Health knowledge and livelihood experiences with COVID-19 amongst Arizona residents
Source: Front Public Health. 2022 Oct 17;10:939154. doi: 10.3389/fpubh.2022.939154 (PMC9618962; doi:10.3389/fpubh.2022.939154)
Supplement: Supplementary file 1 [file Data_Sheet_1.docx]

**APPENDIX 1: QUESTIONNAIRE**

1. Have you heard about the Coronavirus or COVID-19?

Yes

No

2. How would you consider your level of information about what COVID-19?

Very Well

Well

Normal

Not informed

3. What is your main source of information regarding COVID-19?

Radio

Television

Social media

Family/Friends/Neighbors

Other ………….

4. How serious do you feel the current situation is?

Very serious

Not that serious

Not serious at all

I don’t believe this is true

5. Have you experienced in the last two weeks any of these conditions? (mark all relevant ones)

Fever (above 98.6 F or 37 C) Sore Throat

Dry Cough

Chest Pain

Headache

Diarrhea

Joints Pain

Vomiting

Body Aches

Difficulty in Breathing

None of the above (skip to question 6)

5.1. Did you visit during the last two weeks a hospital or clinic because any of these conditions?

Yes

No

6. Have you been tested for COVID-19?

Yes

No (skip to question 7)

6.1 What was the result of your test?

Positive

Negative

I have not received the results yet

7. Has any member of your family, friend, coworker, or neighbor been diagnosed COVID-19 positive?

Yes

No

8. Did you lose your job due to the COVID-19 outbreak?

Yes

No

9. Did any members of your family lose their jobs because of COVID-19?

Yes

No

10. Since the beginning of the COVID-19 outbreak, have you or your family experienced:

(Check all corresponding ones)

Lack of Food

Lack of Hygiene Products

Lack/shortage of income

Other: ……………..

None of the Above

11. Since the beginning of the COVID-19 outbreak, have you visited any schools or food banks to get food for you and your family?

Yes

No

12. Since the start of the COVID-19 outbreak, have you used any of your state, county, or city government programs to support the challenges that have arisen?

Yes

No

13. What preventive measures are taken in your home? (Check all the corresponding ones)

Staying at home for most of the time Wearing a mask when I’m out of the house

Keep 6 feet away from others

people outside the house

Using chlorine/alcohol to clean common areas at home

Washing my hands frequently for 20 seconds

Using 60% and higher alcohol gel to sanitize your hands

Avoiding contact with others if possible

Avoid touching your eyes, mouth, and nose

None of the Above

14. How are you doing today compared to the way you felt in February, before the pandemic? (Check all the ones that apply)

More stressed

More isolated

More anxious

More tired

More sad

More worried about the future

More lonely

None of the Above

15. Have you heard about the COVID-19 vaccine?

Yes

No (poll ends)

16. Where did you hear the information?

Radio, which station?

Television, what station?

Social media, which one?

Family/friends/neighbors

Other, which one? ……………….

17. Have you already received the vaccine against COVID-19?

Yes (continue to question 17.1)

No (continue to question 18)

17.1 What vaccine did you receive?

Pfizer

Moderna

Johnson&Johnson

(Yes, for Pfizer or Moderna, follow up with question 17.2)

(Yes, answered for Johnson & Johnson, ends questionnaire)

17.2 Have you already received the second dose for Pfizer or Moderna?

Yes (ends the questionnaire)

No (continue with 17.3)

17.3 Why have you not received second dose?

I was denied the dose

I don't know where to apply for it

There was none in stock

I didn't go back for personal reasons

Other ………………….

18. Why haven't you received the vaccine against COVID-19?

It still doesn't correspond to me because of age

Lack of online registration

Distrust of the government

Problems with the English language

Lack of transportation

Other ………
